# Epidemiologic analysis of salivary gland tumors over a 10-years period diagnosed in a northeast Brazilian population

**DOI:** 10.4317/medoral.23532

**Published:** 2020-05-10

**Authors:** John Lennon Silva Cunha, Ana Carolina Penha Coimbra, João Vitor Rocha Silva, Ilmara Silva do Nascimento, Maria Eliane de Andrade, Clauberto Rodrigues de Oliveira, Oslei Paes de Almeida, Ciro Dantas Soares, Sílvia Ferreira de Sousa, Ricardo Luiz Cavalcanti de Albuquerque-Júnior

**Affiliations:** 1 DDS, MSc student. Oral Pathology Section, Department of Oral Diagnosis, Piracicaba Dental School, University of Campinas (UNICAMP), SP, Brazil; 2 MD. Department of Medicine, Federal University of Sergipe (UFS), Aracaju, Brazil; 3 Department of Dentistry, Tiradentes University (UNIT), Aracaju, Sergipe, Brazil; 4 PhD student. Laboratory of Morphology and Experimental Pathology, Institute of Technology and Research, Tiradentes University (UNIT), Aracaju, Sergipe, Brazil; 5 DDS, PhD, Professor. Oral Pathology Section, Department of Oral Diagnosis, Piracicaba Dental School, University of Campinas (UNICAMP), SP, Brazil; 6 DDS, PhD student. Oral Pathology Section, Department of Oral Diagnosis, Piracicaba Dental School, University of Campinas (UNICAMP), SP, Brazil; 7 DDS, PhD, Professor. Department of Oral Surgery and Pathology, School of Dentistry, Universidade Federal de Minas Gerais (UFMG), Belo Horizonte, Brazil; 8 DDS, PhD, Professor. Department of Dentistry, Tiradentes University (UNIT), Aracaju, Sergipe, Brazil

## Abstract

**Background:**

Salivary gland tumors (SGT) correspond to a heterogeneous group of lesions with variable biological behavior. The present study aimed to determine the distribution and demographic findings of salivary gland neoplasms in a northeast Brazilian population.

**Material and Methods:**

A retrospective descriptive cross-sectional study was performed. A total of 588 cases of SGT were diagnosed between 2006 and 2016 of 4 pathology services in the state of Sergipe, Brazil. All cases were reviewed, and data such as sex, age, anatomical location, and histopathological diagnosis were collected.

**Results:**

A total of 470 (79.9%) tumors were benign and 118 (20.1%) were malignant. The majority of the patients were females (n=328, 55.8%) with an overall female:male ratio of 1.2:1. The major salivary glands were affected more than the minor glands (69.5% vs. 30.5%). Pleomorphic adenoma (n=419, 71.3%) and mucoepidermoid carcinoma (n=29, 4.9%) were the most frequent benign and malignant tumors, respectively. In addition, both benign and malignant tumors occurred more frequently in the parotid gland (n=300, 51%, *p*<0.05).

**Conclusions:**

The epidemiologic profile and clinical characteristics of SGT were similar to those described in other countries and other regions of Brazil. Epidemiological studies of SGT help to understand their clinical and pathological features and are essential to establish the proper management and prognosis.

** Key words:**Salivary gland, tumors, epidemiology, head and neck pathology.

## Introduction

Salivary gland tumors (SGT) are uncommon lesions that present a wide variation in relation to the clinical, histological, and biological aspects (1,2). In addition, these lesions often represent a diagnostic challenge for the pathologist due to the overlapping of morphological findings (2,3).

SGT account for about 3 to 6% of all tumors in the head and neck region, with an annual estimated global incidence ranging from 0.4 to 13.5 cases per 100,000 individuals (2,4). Although several studies evaluate the frequency and incidence of these tumors in Brazil (1,2,5-10) and other countries of the world (11-16), the epidemiological data of these lesions is not well established because there is a wide variation in the incidence and prevalence of these tumors across countries, indicating a geographic variation in the frequency of these neoplasms (2,9).

Thus, the objective of the present study was to describe the demographic and clinical aspects of salivary gland neoplasms diagnosed in 4 reference pathology centers in the state of Sergipe (Aracaju, Brazil), and to compare the findings with epidemiological data from different geographic locations.

## Material and Methods

- Study design

In this study, the files of 4 surgical pathology centers in Aracaju, Sergipe State, Brazil were retrospectively reviewed: Laboratory of Surgical Pathology of the University Hospital of the Federal University of Sergipe (HU-UFS), Oral Pathology Service of the Tiradentes University (UNIT), and two private general pathology services. During a 10-year period, between January 2006 and December 2016, 588 cases of salivary gland neoplasms were retrieved from these archives.

- Sample

All cases of salivary gland tumors were retrieved, and data such as gender, age, anatomical location, and histopathological diagnosis were obtained from clinical records and analyzed. The lesions were classified into benign and malignant tumors in accordance with the current WHO classification of the head and neck tumors (17). Microscopical slides of all cases were examined by two independent pathologists with more than 25 years of experience. Immunohistochemical and histochemical analyses were performed when routine staining (hematoxylin-eosin) was not sufficient to establish the final diagnosis.

- Analysis

Descriptive and quantitative data analysis was performed using the Statistical Package for the Social Sciences for Windows 20.0 (SPSS, Inc., Chicago, IL, USA). Continuous variables were expressed as mean, median and standard deviation values. Categorical variables were expressed as absolute number of cases and percentage values. Person’s chi-square test and Fisher’s exact test were used to evaluate association between biological behavior (malignant vs benign tumors) and clinical and demographic characteristics, adopting a *p-value* of ≤0.05 and a 95% confidence interval.

## Results

In a 10-year period [2006-2016], there were 588 salivary gland neoplasms diagnosed at the 4 pathology reference centers in Aracaju, Sergipe State, Brazil. Of the total of 588 cases of salivary gland neoplasms, 470 (79.9%) were benign and 118 (20.1%) malignant neoplasms with a benign:malignant ratio of 3.9:1, distributed among 7 benign and 10 malignant histologic subtypes (Table 1).

The majority of patients were female (n=328, 55.8%) with an overall female:male ratio of 1.2:1 (Table 1). Most tumors occurred in the patients between the third and seventh decades of life, with a mean age of 57.9 years (range 2-106 years). The distribution of each salivary gland neoplasm, according to the age of patients, is showed in Table 2.

**Figure 1:**
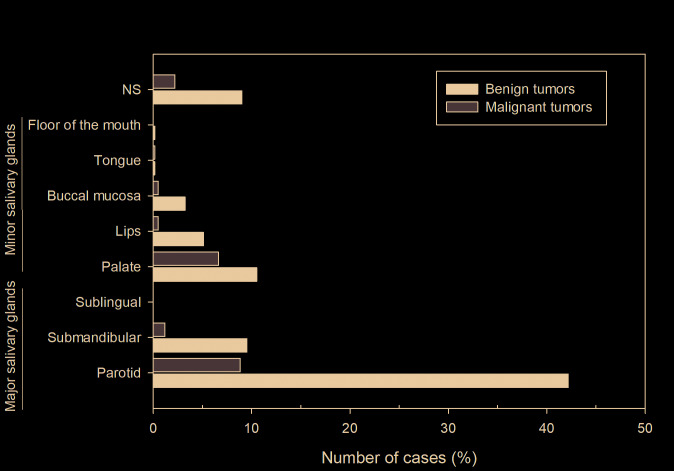
Distribution of 588 salivary gland tumors according to the primary site of involvement. NS, not specified.

Regarding the anatomical site, 69.5% of the tumors occurred in the major salivary glands (n=363, 69.5%) while only 30.5% affected the minor salivary glands. The parotid gland was the most commonly affected, with a frequency of 51% (n=300), followed by the palate (n=101, 17.2%), submandibular gland (n=63, 10.7%), lips (n=33, 5.6%), buccal mucosa (n=22, 3.7%), and floor of the mouth (n=1, 0.2%). There were 66 cases with unspecified anatomic location (11.2%), and none tumor affected the sublingual gland (Fig. 1). Both benign and malignant neoplasms predominated in the parotid gland, followed by the palate and submandibular gland, respectively (Table 3).

**Table 1:**
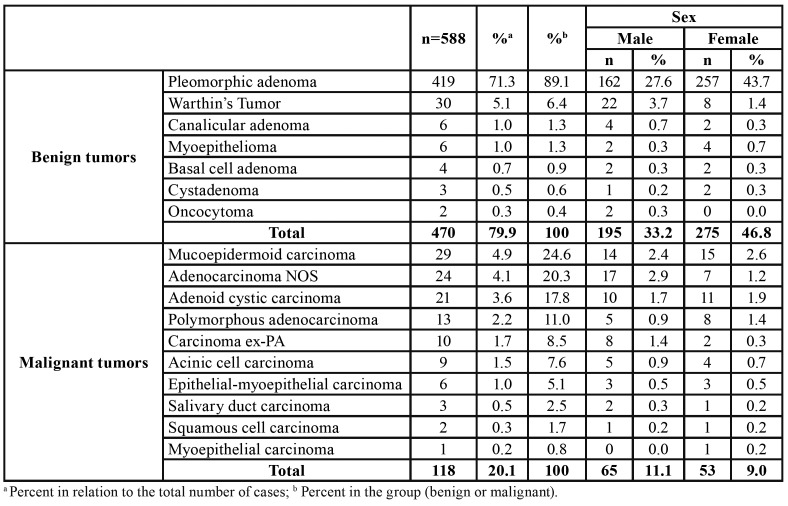
Histologic and gender distribution of 588 benign and malignant neoplasms of salivary glands.

**Table 2:**
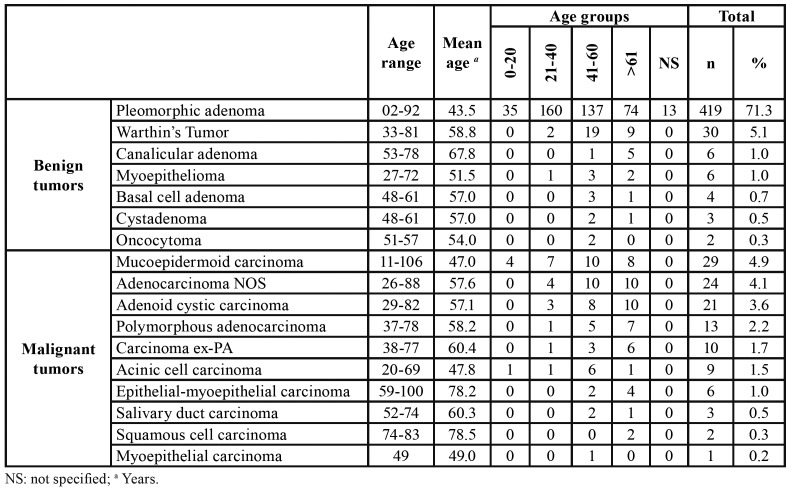
Age group distribution (decade of life) of benign and malignant salivary gland tumors.

**Table 3:**
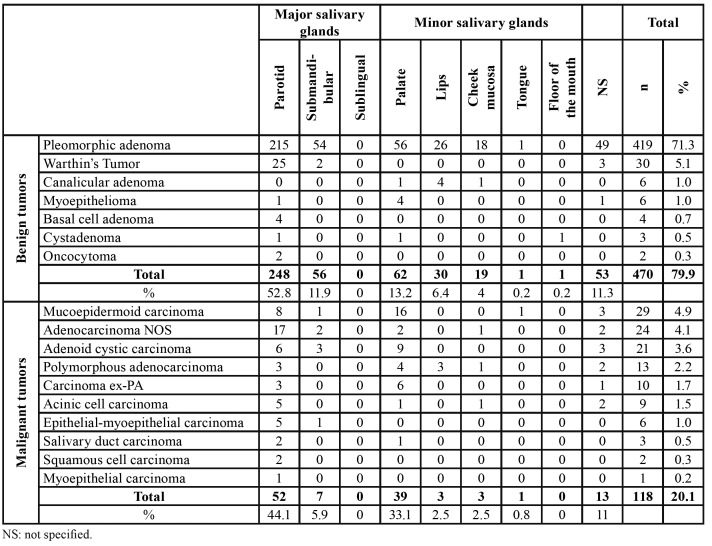
Distribution of the 588 salivary gland tumors according to the location (major and minor salivary glands).

**Figure 2:**
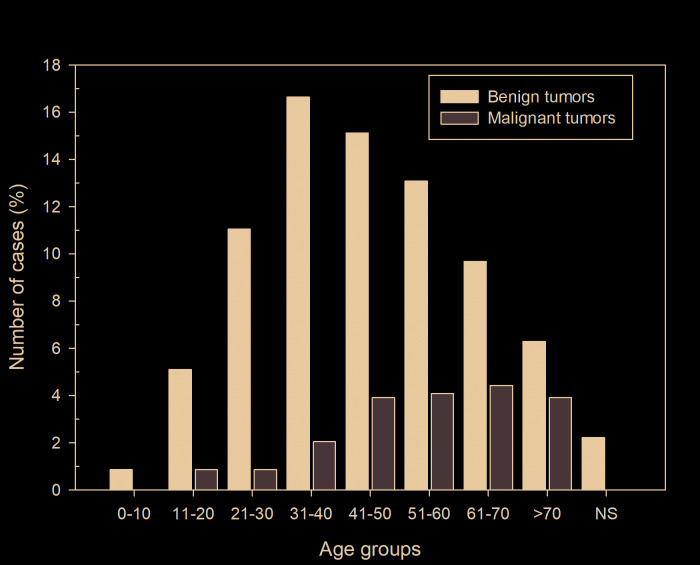
Distribution of 588 salivary gland tumors according to the age group (decade of life).

Among the benign salivary gland tumors, pleomorphic adenoma (PA) was most frequent (n=419; 89.1%) followed by Warthin's tumor (n=30, 6.4%), canalicular adenoma (n=6, 1.3%) and myoepithelioma (n=6, 1.3%) (Table 1). These tumors were diagnosed mainly between the fourth and fifth decades of life (Fig. 2); however, the age ranged from 2 to 92 years, with an average age of 55.7 years (SD±7.4) (Table 2).

Most cases occurred in the parotid gland (n=248, 52.8%) and female patients (n=275; 58.5%), with a female:male ratio of 1.4:1 (275 female and 195 male). Regarding the malignancies, mucoepidermoid carcinoma was the most frequent malignant tumor (n=29, 24.6%), followed by adenocarcinomas not otherwise specified (n=24, 20.3%), and adenoid cystic carcinoma (n=21, 17.8%) (Table 1). The age ranged from 11 to 106 years, with a mean age of 59.4 years (SD±11.1) (Table 2). Most cases also occurred in the parotid gland (n=52, 44.1 %) and male (n=65; 55.1%), with a female:male ratio of 0.8:1.0 (53 female and 65 male) (Table 3).

When the behavior of the tumors (malignant vs benign tumors) was evaluated, the parotid was the most affected gland mainly by benign tumors (*p*<0.05). Also, the benign salivary gland tumors were more common in female patients (*p*<0.05); results were statistically significant (Table 4).

**Table 4:**
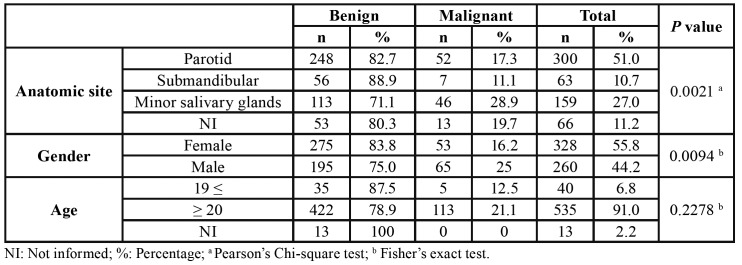
Anatomic site, gender, and age group distribution of benign and malignant salivary gland tumors.

## Discussion

**Figure 3:**
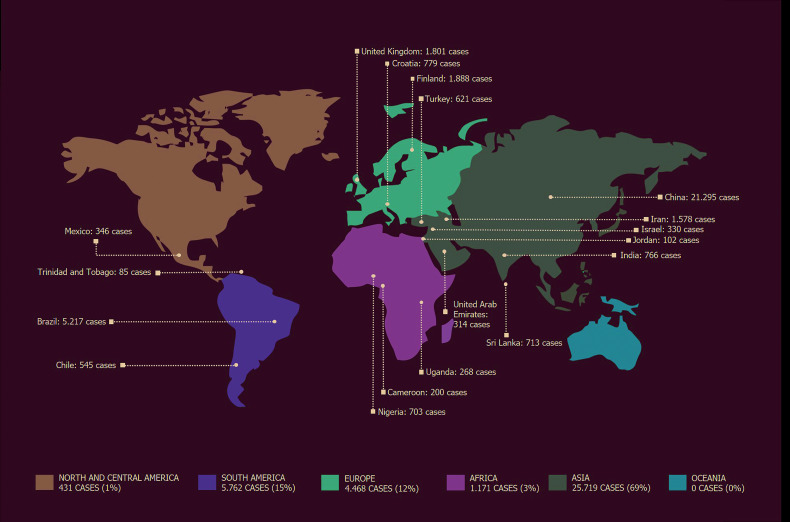
World distribution of 37.205 cases of SGT reported in the literature in the last 20 years [1999-2019].

In the last two decades [1999-2019] several studies performed worldwide have been published on the epidemiology of salivary gland neoplasms, as shown in Fig. 3. According to the WHO [2017], overall, female patients are slightly more affected by salivary gland neoplasms than male patients (17). However, some variations can be found when analyzing specific tumor subtypes (2,9,17,18). In the present study, the female-to-male ratio was 1.2:1, which is in agreement with most studies (19,20), including Brazilian reports (2,6,8,9).

In addition, benign neoplasms presented a male-to-female ratio of 1:1.4, while malignant neoplasms demonstrated a male-to-female ratio of 1:0.8, indicating that benign tumors were more common in female patients, whereas malignancies were slightly more common in males. These data are in accordance with several previous studies (2,9). However, a previous study performed in Mexico showed that female patients were more affected by malignant neoplasms than male patients (21).

In the present study, most tumors were benign (79.9%), data similar to other studies where these tumors correspond to about 51.5 to 86.4% of all salivary gland neoplasms (2,5-9,13). However, some studies conducted on the African (22,23) and Asian continents (24,25) have shown a higher incidence of malignancies, and suggest geographic variation in the frequency of these tumors.

Regarding the benign neoplasms, pleomorphic adenoma was the most common tumor in this study, accounting for 89.1% of all benign neoplasms followed by Warthin’s Tumor (6.4%). In fact, pleomorphic adenoma is the most common benign neoplasm in all previously published studies (1,2,4,5-16,18-30), and Warthin's Tumor was the second benign tumor more frequent (1,5,6,8-10). However, some studies have shown basal cell adenoma (2,26) or myoepithelioma (23,24,27-30) as the second most common benign tumors.

On the other hand, Silva *et al*. (2) performed a retrospective multicentric study in Brazil and observed that basal cell adenoma and cystadenoma were the second and third most common neoplasms, respectively, different from most studies published in Brazil that shows the Whartin’s Tumor as the second most common benign neoplasm (1,5,6,8-10). These results suggest that multicenter studies can better characterize the heterogeneity of tumors in large territories, such as Brazil, for example, and contribute to the comprehension of epidemiological differences in the population (2). Also, the fact that this study was performed in oral pathology services may explain this apparent difference. The Warthin’s Tumor is a neoplasm that affects almost exclusively the parotid gland, and some studies show that most cases from surgical pathology centers affect the major salivary glands, particularly the parotid gland, whereas tumors of the minor salivary glands represent the majority of cases diagnosed in oral pathology services (9). In addition, some other benign tumors, such as oncocytomas, were diagnosed in our study (n = 2, 0.3%). However, these tumors are very rare and are usually observed only in large sample studies (2).

The most common malignant tumor was the mucoepidermoid carcinoma, accounting for 24.6% of the cases, followed by adenocarcinomas not otherwise specified (AcNOS), which represented 20.3%, and cystic adenoid carcinoma (17.8%), that corroborate with previous studies (12-15,19,24-26,29,30). On the other hand, other studies indicate cystic adenoid carcinoma as the most frequent malignant tumor (1,4,7,8,11,21-23,28). In general, the four most frequent malignant tumors are mucoepidermoid carcinoma, cystic adenoid carcinoma, acinar cell carcinoma, and AcNOS.

The morphological diagnosis of salivary gland tumors is challenging due to a large number of histological subtypes, overlapping of morphological findings, and different classifications (2,3). The diagnosis of polymorphous adenocarcinoma, in particular, can be difficult, especially in pathology centers without an experienced pathologist in oral and maxillofacial lesions, since this tumor shares some morphological characteristics with several other tumors (2). In our study, 13 cases of polymorphous adenocarcinomas were diagnosed, of which 8 affected minor intraoral salivary glands, 3 affected parotid glands, and 3 cases with unspecified anatomical sites. This strong predilection for polymorphic adenocarcinoma by minor salivary glands, especially in the palate region, is well established in the literature (17). Furthermore, until the last WHO classification, polymorphous adenocarcinoma was called "low-grade polymorphous adenocarcinoma", because in most cases, it exhibits indolent behavior. However, approximately 10% to 33% of patients develop local recurrences, 9% to 15% have nodal metastases, and some cases are extremely aggressive, with imprecise clinical behavior (2). Considering the variation in the biological behavior of these lesions, the new classification proposed by the WHO for salivary gland neoplasms abandoned the term "low grade" and renamed these tumors only as polymorphous adenocarcinoma (2,17). The purpose of this modification is to avoid possible terminological confusion and to facilitate the choice of treatment, especially for the most unusual cases (2,17). In addition, in our study, some other malignant tumors were very rare, such as salivary duct carcinomas (n=3, 0.5%) and myoepithelial carcinomas (n=1, 0.2%). Although these entities are well recognized, they are also rarely reported in studies with small samples (2).

Regarding the anatomical location, most of the SGTs of this study were diagnosed in the parotid gland, followed by the minor salivary glands of the palate and submandibular gland. In general, this result was also reported by other studies (1,4,9,11,14,15,19,20,23,26). However, some studies have shown that malignant neoplasms preferentially affect the minor intraoral salivary glands (1,2,9).

In summary, although several studies evaluate the frequency and incidence of salivary gland neoplasms, continuous studies that report the incidence and characteristics of these lesions are essential to keep physicians and surgeons up to date, especially when the classification of these tumors undergoes some change (2).

## Conclusions

The results of this study were similar to those found by several other authors in Brazil and worldwide. The pleomorphic adenoma was the most common benign tumor, and the mucoepidermoid carcinoma the most frequent malignant tumor in the salivary glands. In addition, both benign and malignant tumors occurred more frequently in the parotid gland.

## References

[1] Vasconcelos AC, Nör F, Meurer L, Salvadori G, Souza LB, Vargas PA (2016). Clinicopathological analysis of salivary gland tumors over a 15-year period. Braz Oral.

[2] da Silva LP, Serpa MS, Viveiros SK, Sena DAC, de Carvalho Pinho RF, de Abreu Guimarães LD (2018). Salivary gland tumors in a Brazilian population: A 20-year retrospective and multicentric study of 2292 cases. J Craniomaxillofac Surg.

[3] Rooper LM (2019). Challenges in Minor Salivary Gland Biopsies: A Practical Approach to Problematic Histologic Patterns. Head Neck Pathol.

[4] Tian Z, Li L, Wang L, Hu Y, Li J (2010). Salivary gland neoplasms in oral and maxillofacial regions: a 23-year retrospective study of 6982 cases in an eastern Chinese population. Int J Oral Maxillofac Surg.

[5] Vargas PA, Gerhard R, Araújo Filho VJ, de Castro IV (2002). Salivary gland tumors in a Brazilian population: a retrospective study of 124 cases. Rev Hosp Clin Fac Med Sao Paulo.

[6] Ito FA, Ito K, Vargas PA, de Almeida OP, Lopes MA (2005). Salivary gland tumors in a Brazilian population: a retrospective study of 496 cases. Int J Oral Maxillofac Surg.

[7] Lima SS, Soares AF, Amorim RFB, Freitas RA (2005). Epidemiologic profile of salivary gland neoplasms: analysis of 245 cases. Bras Otorrinolaringol.

[8] de Oliveira FA, Duarte EC, Taveira CT, Máximo AA, de Aquino EC, Alencar Rde C (2009). Salivary gland tumor: a review of 599 cases in a Brazilian population. Head Neck Pathol.

[9] Fonseca FP, Carvalho M de V, de Almeida OP, Rangel AL, Takizawa MC, Bueno AG (2012). Clinicopathologic analysis of 493 cases of salivary gland tumors in a Southern Brazilian population. Oral Surg Oral Med Oral Pathol Oral Radiol.

[10] Bittar RF, Ferraro HP, Moraes Gonçalves FT, Couto da Cunha MG, Biamino ER (2015). Neoplasms of the salivary glands: analysis of 727 histopathological reports in a single institution. Otolaryngol Pol.

[11] Bello IO, Salo T, Dayan D, Tervahauta E, Almangoush A, Schnaiderman-Shapiro A (2012). Epithelial salivary gland tumors in two distant geographical locations, Finland (Helsinki and Oulu) and Israel (Tel Aviv): a 10-year retrospective comparative study of 2,218 cases. Head Neck Pathol.

[12] Wang YL, Zhu YX, Chen TZ, Wang Y, Sun GH, Zhang L (2012). Clinicopathologic study of 1176 salivary gland tumors in a Chinese population: experience of one cancer center 1997-2007. Acta Otolaryngol.

[13] Bradley PJ, McGurk M (2013). Incidence of salivary gland neoplasms in a defined UK population. Br J Oral Maxillofac Surg.

[14] Wang XD, Meng LJ, Hou TT, Huang SH (2015). Tumours of the salivary glands in northeastern China: a retrospective study of 2508 patients. Br J Oral Maxillofac Surg.

[15] Gao M, Hao Y, Huang MX, Ma DQ, Chen Y, Luo HY (2017). Salivary gland tumours in a northern Chinese population: a 50-year retrospective study of 7190 cases. Int J Oral Maxillofac Surg.

[16] Campolo González A, Ramírez Skinner H, Vargas Díaz A, León Ramírez A, Goñi Espildora I, Solar González A (2018). Epithelial tumors of salivary glands. Review of 286 pathology reports. Rev Med Chil.

[17] Seethala RR, Stenman G (2017). Update from the 4th Edition of the World Health Organization Classification of Head and Neck Tumours: Tumors of the Salivary Gland. Head Neck Pathol.

[18] Noel L, Medford S, Islam S, Muddeen A, Greaves W, Juman S (2018). Epidemiology of salivary gland tumours in an Eastern Caribbean nation: A retrospective study. Ann Med Surg (Lond).

[19] Li LJ, Li Y, Wen YM, Liu H, Zhao HW (2008). Clinical analysis of salivary gland tumor cases in West China in past 50 years. Oral Oncol.

[20] Araya J, Martinez R, Niklander S, Marshall M, Esguep A (2015). Incidence and prevalence of salivary gland tumours in Valparaiso, Chile. Med Oral Patol Oral Cir Bucal.

[21] Mejía-Velázquez CP, Durán-Padilla MA, Gómez-Apo E, Quezada-Rivera D, Gaitán-Cepeda LA (2012). Tumors of the salivary gland in Mexicans. A retrospective study of 360 cases. Med Oral Patol Oral Cir Bucal.

[22] Fomete B, Adebayo ET, Ononiwu CN (2015). Management of salivary gland tumors in a Nigerian tertiary institution. Ann Afr Med.

[23] Lawal AO, Adisa AO, Kolude B, Adeyemi BF, Olajide MA (2013). A review of 413 salivary gland tumours in the head and neck region. J Clin Exp Dent.

[24] Taghavi N, Sargolzaei S, Mashhadiabbas F, Akbarzadeh A, Kardouni P (2016). Salivary Gland Tumors: A 15- year Report from Iran. Turk Patoloji Derg.

[25] Tilakaratne WM, Jayasooriya PR, Tennakoon TM, Saku T (2009). Epithelial salivary tumors in Sri Lanka: a retrospective study of 713 cases. Oral Surg Oral Med Oral Pathol Oral Radiol Endod.

[26] Jones AV, Craig GT, Speight PM, Franklin CD (2008). The range and demographics of salivary gland tumours diagnosed in a UK population. Oral Oncol.

[27] Otoh EC, Johnson NW, Olasoji H, Danfillo IS, Adeleke OA (2005). Salivary gland neoplasms in Maiduguri, north-eastern Nigeria. Oral Dis.

[28] Vuhahula EA (2004). Salivary gland tumors in Uganda: clinical pathological study. Afr Health Sci.

[29] Torabinia N, Khalesi S (2014). Clinicopathological study of 229 cases of salivary gland tumors in Isfahan population. Dent Res J (Isfahan).

[30] Saghravanian N, Ghazi N, Saba M (2013). Clinicopathologic evaluation of salivary gland neoplasms: a 38-year retrospective study in Iran. Ann Diagn Pathol.

